# Analysis of *ZAP70* expression in adult acute lymphoblastic leukaemia by real time quantitative PCR

**DOI:** 10.1186/1755-8166-5-22

**Published:** 2012-05-01

**Authors:** Geothy Chakupurakal, Andrew Bell, Mike Griffiths, Farooq Wandroo, Paul Moss

**Affiliations:** 1 School of Cancer Sciences, University of Birmingham, Birmingham, UK; 2 West Midlands Regional Genetics Laboratory, Birmingham Women’s NHS Foundation Trust, Birmingham B15 2TG, UK; 3 Sandwell and West Birmingham Hospitals NHS Trust, Birmingham B18 7QH, UK

**Keywords:** ZAP70, ALL, RT-qPCR

## Abstract

**Background:**

*ZAP70* gene expression is associated with poor prognosis in B-cell lymphoproliferative disorders especially chronic lymphocytic leukaemia (CLL) but its role in adult B-ALL has not been established. On diagnostic samples from 76 patients with adult ALL (65 with B-ALL and 11 with T-ALL) *ZAP70* mRNA expression levels were studied by real time-quantitative PCR (RT-qPCR) analysis.

**Findings:**

A broad distribution of *ZAP70* expression was observed in ALL, ranging from 0.002 to 5.3 fold that of the *ZAP70* positive Jurkat reference cell line. No association was observed between expression levels and the presence of specific cytogenetic abnormalities. Five cases, including one case of T-ALL, had *ZAP70* expression above the level of the Jurkat reference cell line.

**Conclusions:**

Our results confirm the frequent expression of *ZAP70* in adult ALL. Limited comparisons made did highlight poor-risk patients with high *ZAP70* expression, but due to lack of clinical information on patient samples we were unable to directly assess the impact on disease prognosis. *ZAP-70* may be an important laboratory assay in adult ALL and further studies are warranted to study a potential correlation with cytogenetic and other genetic markers.

## Background

Zeta associated protein tyrosine kinase (*ZAP70*) is a 70kD molecule associated with the ζ-chain of the CD3 T-cell receptor (*TCR*) complex [1]. On formation of the immunological synapse, immune receptor tyrosine-based activation motifs (ITAM) in the CD3 and ζ-chains are activated by phosphorylation. The ζ-chains become docking sites for *ZAP70* thereby activating the MAP kinase, calcium/calcineurin and protein kinase C signalling pathways. Thus *ZAP70* plays a major part in lymphocyte signal transduction resulting in cell differentiation and proliferation [2].

*ZAP70* gene expression plays a critical role in the transition of pre-B to pro-B cells within the bone marrow. Genetic inactivation of the *ZAP70* gene results in failure of pre-B cell receptor (pre-BCR) induced differentiation, proliferation and heavy chain exclusion, and a failure to progress beyond the pre-B cell stage [3]. *ZAP70* is not expressed in normal mature B cells derived from bone marrow, peripheral blood, or tonsil [4]. *ZAP70* expression is also observed in a large proportion of patients with chronic lymphocytic leukaemia (CLL) where it has been associated with poor clinical outcome in several studies [5]. More recently, *ZAP70* protein has been detected in a wide variety of additional B-cell lymphoproliferative disorders including mantle cell lymphoma, diffuse large B-cell lymphoma, and Burkitt lymphoma [6,7].

While several groups, including our own, have demonstrated *ZAP70* mRNA expression in paediatric pre-B ALL [8], there have been no previous studies that have used RT-qPCR to determine *ZAP70* expression in adult cases of ALL and then correlated the results with cytogenetic abnormalities at diagnosis. In the present work we have used RT-qPCR analysis to determine the expression of *ZAP70* mRNA levels in 76 adult patients with B-ALL or T-ALL at the time of diagnosis. We show that *ZAP70* gene expression is detected in almost all cases with a broad range of expression levels across the cohort. No association was observed between *ZAP70* expression and cytogenetic abnormalities identified in these patients.

## Results

**Figure 1:**
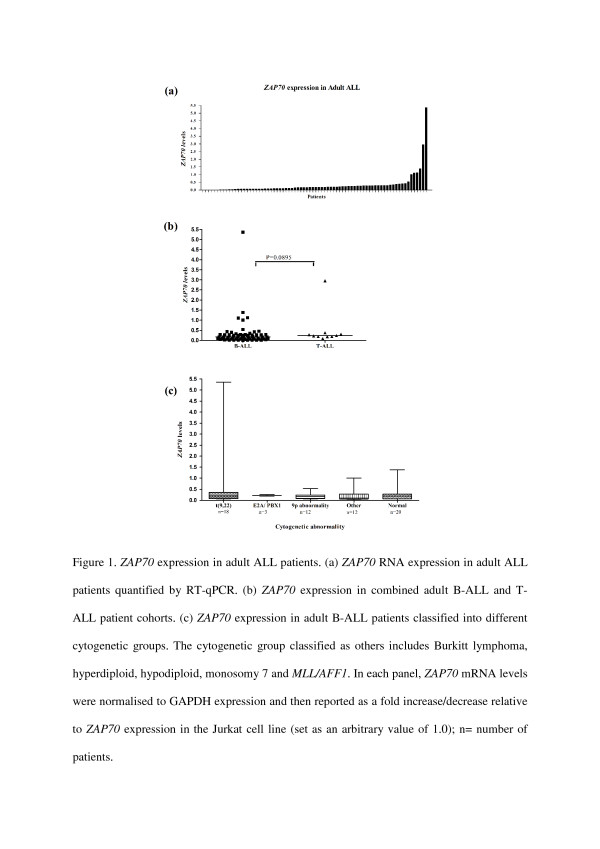
***ZAP70*expression in adult ALL patients. (a)***ZAP70* RNA expression in adult ALL patients quantified by RT-qPCR. **(b)***ZAP70* expression in combined adult B-ALL and T-ALL patient cohorts. **(c)***ZAP70* expression in adult B-ALL patients classified into different cytogenetic groups. The cytogenetic group classified as others includes Burkitt lymphoma, hyperdiploid, hypodiploid, monosomy 7 and *MLL/AFF1*. In each panel, *ZAP70* mRNA levels were normalised to GAPDH expression and then reported as a fold increase/decrease relative to *ZAP70* expression in the Jurkat cell line (set as an arbitrary value of 1.0); n = number of patients.

In total we examined 76 cases of ALL, of which 65 were of B-cell origin (B-ALL) and 11 of T-cell origin (T-ALL). *ZAP70* mRNA expression was measured as fold increase/decrease relative to expression levels in the reference *ZAP70* positive Jurkat cell line Table 1. A broad range of distribution of *ZAP70* mRNA expression (range 0.002-5.36; median 0.169; mean 0.306) (Figure 1a) was observed. As expected, *ZAP70* expression was seen in all cases of T-ALL (range 0.09-2.955; median 0.233; mean 0.504) (Figure 1b). Overall, 5 cases (4 B-ALL, 1 T-ALL) expressed *ZAP70* at higher levels than the reference *ZAP70* positive Jurkat cell line (Figure 1b).

**Table 1:** Patient demographic and cytogenetic data

**Type**	**Cytogenetics**	**Cases**	**M/F**	**Age**^**a**^	***ZAP70 *expression**^**b**^	**Associated abnormalities**
T-ALL		11	6/5	44.7; (19-56)	0.363; 0.252; (0.090-2.955)	9p abnormality(n = 5) 6q deletion (n = 2 t(10;14) (n = 1)
B-ALL		65	36/29	46.0; (18-79)	0.332; 0.185; (0.002-5.360)	
B-ALL	t(9;22)	18	10/8	44.4; (23-70)	0.440; 0.191; (0.010-5.360)	Hyperdiploid (n = 2), Monosomy 7 (n = 2) 9p abnormality (n = 3) 12p gain (n = 2) t(1;19) (n = 1)
B-ALL	t(1;19) *TCF3-PBX1*	3	2/1	43.3; (42-52)	0.219; 0.210; (0.193-0.254)	9p abnormality (n = 2) 6q deletion (n = 2)
B-ALL	9p abnormality	12	8/4	45.2; (18-72)	0.180; 0.176; (0.004-0.535)	Monosomy7 (n = 2) RUNX1 (n = 2) 12p abnormality (n = 3) 6q deletion (n = 2)
B-ALL	Burkitt lymphoma	6	3/3	44.7; (22-78)	0.159; 0.168; (0.005-0.350)	12p abnormality (n = 1) 9p abnormality (n = 2)
B-ALL	Hyperdiploid	6	5/1	45.9; (22-76)	0.232; 0.184; (0.004-1.383)	
B-ALL	Monosomy 7	4	1/3	44.9; (37-58)	0.177; 0.182; (0.015-0.399)	12p abnormality (n = 1)
B-ALL	Hypodiploid	3	1/2	43.7; (18-79)	0.173; 0.182; (0.067-0.290)	12p abnormality (n = 1)
B-ALL	MLL/AFF1	1	1	30.0	0.089	12p abnormality
B-ALL	Normal 46, XX or 46, XY	12	6/6	46.7; (18-82)	0.140; 0.100; (0.002-0.291)	

^a^ Mean patient age in years (range).

^b^ ZAP70 RNA expression was quantified by RT-qPCR and the normalised values expressed relative to that seen in Jurkat control cells. Data shown are mean, median and range for each sub-group.

We then searched for potential correlations between *ZAP70* expression and known genetic abnormalities within the B-ALL tumours. 53/65 (82%) in the B-ALL group had cytogenetic changes such as t(9;22) (n = 18), 9p abnormality (n = 12), t(1;19) *TCF3-PBX1* gene fusion, (previously called *E2A-PBX1*) (n = 3) and a range of other abnormalities, including Burkitt lymphoma, hyperdiploidy, hypodiploidy, monosomy 7 and 12p abnormality (n = 20). 12 cases of B-ALL had no observable cytogenetic abnormality. No association was observed between the level of *ZAP70* expression and individual cytogenetic subgroups (Figure 1c), although we noted a statistically insignificant trend towards increased levels of *ZAP70* mRNA in cases with monosomy 7 and 12p abnormalities (data not shown). No association was found between *ZAP70* mRNA expression and the *ZAP70* copy numbers based on cytogenetic data ( Additional file1).

While a relatively continuous distribution pattern of *ZAP70* mRNA levels was seen across the B-ALL cohort, the level of expression was markedly increased in 4 cases, with values ranging from 1.1-5.4 (mean 2.4). Of these B-ALL patients, two had complex cytogenetics whilst the other two had t(9,22) translocations thus predicting poor outcomes in all four cases [9]; however the remaining 16 patients who carried a t(9;22) cytogenetic abnormality had ZAP70 expression levels within the main distribution. Similar to CLL patients where a high *ZAP70* expression level is associated with a poor prognosis, *ZAP70* mRNA expression may be relevant to the prognosis of patients with B-ALL.

## Discussion

This is the first distribution profile for *ZAP70* mRNA expression in adult B-lineage ALL patients by RT-qPCR. The results demonstrate a broad range of expression and a markedly increased expression in a small proportion of cases (6%). Chiaretti et al*.*[10] used microarray analysis to determine *ZAP70* mRNA expression in 95 adult ALL cases followed by immunoblotting to confirm protein expression. In their study, relatively high *ZAP70* expression levels were observed in patients with the t(1;19) *TCF3-PBX1* gene rearrangement but similar high *ZAP70* levels were not seen in our cohort. CDKN2A, a tumour suppressor gene on chromosome 9, can be inactivated by deletion, mutation or methylation. Its role in B-ALL is currently under dispute and has been identified by some groups to have a prognostic role in childhood and adult ALL [11-15]. We analysed patients with 9p abnormalities, without t(9;22), t(1;19), t(8;14), and found no association with *ZAP70* mRNA levels.

Although the biochemical basis for the correlation between *ZAP70* expression and poor prognostic aggressive disease in CLL is unknown, an association with enhanced signal transduction through the pre-BCR complex and phosphorylation of phosphotyrosine phosphatase has been observed [16]. Unfortunately clinical information on patients recruited into this study could not be obtained, though the cytogenetic information on patients with high *ZAP70* mRNA levels suggests possible inferior outcomes. This potential association of high *ZAP70* mRNA levels with inferior outcomes may not be independent, as the cytogenetic findings in the four patients with the highest *ZAP70* expression, i.e. complex or t(9;22), also predict a poor prognosis. However cytogenetic assessment can be difficult [17] and therefore *ZAP70* mRNA expression levels may have a role as an alternative prognostic marker in patients with adult B-ALL. This in turn may allow escalation or de-escalation of therapeutic strategies as well as the possibility of using the *ZAP70*-specific inhibitor, Piceatannol [18] as an adjuvant in ALL therapy. Large cohort studies in adult ALL patients addressing the role of *ZAP70* mRNA expression levels in association with cytogenetic and other genetic markers are hence warranted.

## Methods

### Patient samples

Bone marrow specimens were collected at disease presentation from adult ALL patients attending various centres in West Midlands, UK and referred to the West Midlands Regional Genetics Laboratory between 1998 and 2005, as part of routine genetic analysis of the leukaemias. Bone marrow mononuclear cells (BMMC) were isolated either by Ficoll density centrifugation or by red cell lysis using Erythrocyte Lysis Buffer (Qiagen, Crawley, UK). BMMC were lysed and total RNA was recovered in 60 μl RNAse free water using QIAamp spin columns (QIAamp RNA Blood Mini Kit, Qiagen, Crawley, UK). Excess material after diagnostic testing was stored within the ethically approved Central England Haemato-oncology Research Biobank (REC reference: 09/H0405/12). Anonymised cDNA samples from the biobank were used for the *ZAP70* study. Samples included in the study were from consecutive cases where sufficient excess material remained in patients older than 16.

### Routine cytogenetics, FISH and endpoint RT-PCR

Cytogenetic analysis was performed as part of routine analysis using standard methods (G-banding). FISH analysis for MLL rearrangements, BCR-ABL1 gene fusions, and ETV6-RUNX1 gene fusion was routinely performed on all ALL samples [17], using commercial probes following manufacturer’s protocols (Vysis LSI MLL Dual Color, Break Apart Rearrangement Probe; Vysis LSI BCR/ABL Dual Color, Dual Fusion Translocation Probe; Vysis LSI ETV6(TEL)/RUNX1(AML1) ES Dual Color Translocation Probe Set) (Abbott Molecular, Illinois, USA). In addition, other FISH probes were used variably due to suspicion on G-band analysis and changes in routine FISH screening policies during the period when the samples were collected (Vysis LSI p16 (9p21) SpectrumOrange/CEP 9 SpectrumGreen Probe; MYC Break Apart FISH Probe Kit; IGH/MYC/CEP 8 Tri-Color DF FISH Probe Kit; LSI IGH Dual Color, Break Apart Rearrangement Probe; CEP4 and CEP10 probes (Abbott Molecular, Illinois, USA); and Dako SIL-TAL1 FISH DNA Probe, Sub-Deletion Signal (Dako, Glostrup, Denmark)). End-point RT-PCR for BCR-ABL1 transcripts was performed routinely using standard procedures [19].

### Quantification of *ZAP70* expression by RT-qPCR

Relative quantitation of *ZAP70* mRNA expression was performed using an ABI 7700 Sequence Detection System (Applied Biosystems UK) and analysed using SDS software 1.7. *ZAP70* transcripts were detected using the following primers and probe: forward primer 5'-CGCTGCACAAGTTCCTGGT-3', reverse primer 5'-GACACCTGGTGCAGCAGCT-3', Taqman probe 5'-(FAM)-CATTGCTCACAGGGATCTCCTCCCTCT-(TAMRA)-3'. GAPDH transcripts, which served as an internal control, were quantified using a commercial assay (Applied Biosystems, U.K.). PCR amplification and data normalisation were performed as previously described [8]. These normalised *ZAP70* to *GAPDH* ratios were then calculated as a fold change relative to the *ZAP70* to *GAPDH* ratio of the *ZAP70* positive Jurkat T-cell line, defined as having an expression level of 1.0. All test samples were run in duplicate (mean values used) and template-negative samples served as control and were always negative.

### Statistical analysis

Statistical analysis was performed by group comparison using either a two-sample test or ANOVA and correlation analysis was evaluated with Pearson coefficient, using GraphPad Prism 4 and SPSS software.

## List of abbreviations

ALL, Acute lymphoblastic leukaemia; CLL, Chronic lymphocytic leukaemia; ZAP70, Zeta associated protein70.

## Competing interests

The authors declare no competing interests.

## Author’s contributions

PM and MG conceived and co-ordinated the study. GC and AB carried out molecular genetic studies. GC, AB and MG analysed the data. GC drafted the manuscript. PM, MG and FW participated in study design and helped in drafting the manuscript. All authors read and approved the final manuscript.

## Supplementary Material

Additional file 1 **Table S1.**ZAP70 expression data and cytogenetic analysis of individual ALL samples. Click here for file
