# Supplementary material for: Analysis of ZAP70 expression in adult acute lymphoblastic leukaemia by real time quantitative PCR
Source: Mol Cytogenet. 2012 May 1;5:22. doi: 10.1186/1755-8166-5-22 (PMC3428655; doi:10.1186/1755-8166-5-22)
Supplement: Additional file 1 — Table S1.ZAP70 expression data and cytogenetic analysis of individual ALL samples. [file 1755-8166-5-22-S1.doc]

**Supplementary Table 1**

**ZAP70 expression data and cytogenetic analysis of individual ALL samples.**

| **Patient no** | ***ZAP70* expression^b^** | **ZAP70**  **copy no^a^** | **Major group** | **Associated abnormalities** | **Abnormal FISH and RT-PCR results** |
| --- | --- | --- | --- | --- | --- |
| **1** | 0.002 |  | Normal | Normal |  |
| **2** | 0.004 | 2 | Hyperdiploid | Hyperdiploid Hypodiploid |  |
| **3** | 0.004 | 2 | 9p abnormality | 9p deletion |  |
| **4** | 0.005 | 2 | Burkitt lymphoma | Burkitt t(8;14) |  |
| **5** | 0.010 | 2/2/3/2 | Ph positive | Ph positive  Hyperdiploid | e1a2 BCR-ABL1 by RT-PCR |
| **6** | 0.011 | 2 | Ph positive | Ph positive Monosomy 7 | e1a2 BCR-ABL1 by RT-PCR |
| **7** | 0.014 |  | Normal | Normal |  |
| **8** | 0.015 | 2 | Monosomy 7 | Monosomy 7 |  |
| **9** | 0.027 |  | Normal | Normal |  |
| **10** | 0.032 | 2 | Burkitt lymphoma | Burkitt t(8;14) 9p abnormality |  |
| **11** | 0.048 | 2 | Ph positive | Ph positive | e14a2 BCR-ABL1 by RT-PCR |
| **12** | 0.053 | 3 | Hyperdiploid | Hyperdiploid |  |
| **13** | 0.063 | 2 | 9p abnormality | 9p abnormality |  |
| **14** | 0.064 |  | Normal | Normal |  |
| **15** | 0.067 | 2/4 | Hypodiploid | Hypodiploid Monosomy 7 |  |
| **16** | 0.070 |  | Normal | Normal |  |
| **17** | 0.072 | 2/4/2 | Hypodiploid | Hypodiploid Monosomy 7 12p abnormality |  |
| **18** | 0.072 | 2/2 | Ph positive | Ph positive | e13a2 BCR-ABL1 by RT-PCR |
| **19** | 0.073 | 2 | 9p abnormality | 9p abnormality |  |
| **20** | 0.075 | 2 | Ph positive | Ph positive | e1a2 BCR-ABL1 by RT-PCR |
| **21** | 0.078 | 2/2 | Normal | Normal |  |
| **22** | 0.083 | 2 | Ph positive | Ph positive | e13a2 BCR-ABL1 by RT-PCR |
| **23** | 0.089 | 2 | 9p abnormality | RUNX1 amplification 9p abnormality | RUNX1 (AML1) amplification ETV6 (TEL) deletion |
| **24** | 0.090 | 2 | T-ALL | 9p deletion | Bi-allelic CDKN2A (P16) deletion |
| **25** | 0.097 | 2 | Hyperdiploid | Hyperdiploid | Gain of chromosomes 4 (4 cen), 10 (10 cen) and 21 (RUNX1) |
| **26** | 0.099 | 2 | 9p abnormality | RUNX1 amplification 9p deletion | RUNX1 (AML1) amplification Bi-allelic CDKN2A (P16) deletion |
| **27** | 0.099 | 2 | Monosomy 7 | 6q deletion Monosomy 7 12p deletion |  |
| **28** | 0.105 | 2 | Ph positive | Ph positive Monosomy 7 Hypodiploid | e1a2 BCR-ABL1 by RT-PCR |
| **29** | 0.119 | 2 | Ph positive | Ph positive | e13a2 BCR-ABL1 by RT-PCR |
| **30** | 0.122 | 2 | Monosomy 7 | Monosomy 7 |  |
| **31** | 0.123 | 2/2 | Burkitt lymphoma | Burkitt t(8;14) |  |
| **32** | 0.150 | 4 | Burkitt lymphoma | Burkitt t(8;14) 9p abnormality 12p deletion Hypodiploid |  |
| **33** | 0.165 | 2 | 9p abnormality | 6q deletion Monosomy 7 9p deletion |  |
| **34** | 0.168 | 2/2 | MLL/AFF4 | t(4;11) MLL-AFF4 | MLL gene rearrangement |
| **35** | 0.170 |  | Normal | Normal |  |
| **36** | 0.170 | 2/3 | Ph positive | Ph positive Hyperdiploid | e1a2 BCR-ABL1 by RT-PCR |
| **37** | 0.182 | 2 | 9p abnormality | Monosomy 7 9p abnormality |  |
| **38** | 0.184 | 2 | 9p abnormality | 9p deletion 12p deletion | CDKN2A (P16) deletion ETV6 (TEL) deletion |
| **39** | 0.186 | 2 | T-ALL | 6q deletion |  |
| **40** | 0.189 | 2 | T-ALL | 9p deletion STIL-TAL1 | CDKN2A (P16) deletion STIL deletion |
| **41** | 0.193 | 2 | 9p abnormality, t(1,19) | t(1;19) TCF3(E2A)-PBX1 6q deletion 9p deletion |  |
| **42** | 0.195 | 2 | T-ALL | Normal |  |
| **43** | 0.197 | 2 | 9p abnormality | 9p abnormality |  |
| **44** | 0.200 |  | Normal | Normal |  |
| **45** | 0.210 | 2/2 | 9p abnormality, t(1,19) | t(1;19) TCF3(E2A)-PBX1 6q deletion 9p deletion | CDKN2A (P16) deletion |
| **46** | 0.224 | 2 | T-ALL | 6q deletion |  |
| **47** | 0.234 | 2 | Ph positive | Ph positive | e1a2 BCR-ABL1 by RT-PCR |
| **48** | 0.241 | 2 | T-ALL | 6q deletion 9p deletion 12p abnormality | Bi-allelic CDKN2A (P16) deletion |
| **49** | 0.249 | 2 | Ph positive | Ph positive | e1a2 BCR-ABL1 by RT-PCR |
| **50** | 0.254 | 2 | t(1;19) | Other |  |
| **51** | 0.255 | 2 | 9p abnormality | 9p abnormality |  |
| **52** | 0.265 | 2 | Burkitt lymphoma | Burkitt t(8;14) | IGH@-MYC [8%] |
| **53** | 0.274 |  | Normal | Normal |  |
| **54** | 0.275 |  | Normal | Normal |  |
| **55** | 0.278 | 2 | T-ALL | Normal |  |
| **56** | 0.280 | 2/2/2 | 9p Abnormality | 9p deletion |  |
| **57** | 0.280 | 2 | Normal | Other |  |
| **58** | 0.287 | 2 | Ph positive | Ph positive | e1a2 BCR-ABL1 by RT-PCR |
| **59** | 0.290 | 1 | Hypodiploid | Hypodiploid |  |
| **60** | 0.291 | 2/2 | Normal |  | IGH@ gene rearrangement |
| **61** | 0.296 | 2 | Ph positive | Ph positive 9p deletion | e1a2 BCR-ABL1 by RT-PCR |
| **62** | 0.304 | 2 | T-ALL | 9p deletion | CDKN2A (P16) deletion |
| **63** | 0.333 | 2/2 | Ph positive | Ph positive | e1a2 BCR-ABL1 by RT-PCR |
| **64** | 0.350 | 2 | Burkitt lymphoma | Burkitt t(8;14) |  |
| **65** | 0.375 | 2 | T-ALL | 6q deletion |  |
| **66** | 0.399 | 2 | Monosomy 7 | Monosomy 7 |  |
| **67** | 0.419 | 2 | T-ALL | 9p deletion | Bi-allelic CDKN2A (P16) deletion |
| **68** | 0.435 | 2/2 | Ph positive | Ph positive t(1;19) TCF3(E2A)-PBX1 9p deletion | e14a2 BCR-ABL1 by RT-PCR |
| **69** | 0.455 | 3/3 | Ph positive | Ph positive | e1a2 BCR-ABL1 by RT-PCR |
| **70** | 0.535 | 2 | 9p Abnormality | 9p deletion | Bi-allelic CDKN2A (P16) deletion |
| **71** | 1.006 | 2 | Hyperdiploid | Hyperdiploid |  |
| **72** | 1.101 | 4 | Ph positive | Ph positive 12p abnormality Hyperdiploid | e1a2 BCR-ABL1 by RT-PCR |
| **73** | 1.126 | 2 | Hyperdiploid | Hyperdiploid |  |
| **74** | 1.383 | 2 | Hyperdiploid | Other |  |
| **75** | 2.955 | 2/2 | T-ALL | 9p deletion | Bi-allelic CDKN2A (P16) deletion |
| **76** | 5.360 | 2 | Ph positive | Ph positive | e1a2 BCR-ABL1 by RT-PCR |

^a^ *ZAP70* copy number by karyotype (based on G-band 2q11) in abnormal clones.
